# *Rhodobacter sphaeroides* Extract Lycogen™ Attenuates Testosterone-Induced Benign Prostate Hyperplasia in Rats

**DOI:** 10.3390/ijms19041137

**Published:** 2018-04-10

**Authors:** Chiang-Ting Wang, Ya-Yun Wang, Wen-Sheng Liu, Chun-Ming Cheng, Kuo-Hsun Chiu, Li-Lian Liu, Xue-Zhu Liu, Zhi-Hong Wen, Ya-Huey Chen, Tsung-Ming Chen

**Affiliations:** 1 Department of Marine Biotechnology and Resources, National Sun Yat-sen University, 70 Lienhai Rd., Kaohsiung 80424, Taiwan; wct5816@gmail.com; 2 Department of Medicine, Kaohsiung Armed Forces General Hospital, Kaohsiung 80284, Taiwan; 3 Department of Chemistry, Fu Jen Catholic University, New Taipei City 24205, Taiwan; vic0009@gmail.com; 4 Graduate Institute of Biomedical and Pharmaceutical Science, Fu Jen Catholic University, New Taipei City 24205, Taiwan; 5 Asia-Pacific Biotech Developing, Inc., Kaohsiung 80681, Taiwan; wensheng5394@gmail.com; 6 Department and Graduate Institute of Aquaculture, National Kaohsiung University of Science and Technology, No.142, Haijhuan Rd., Nazih Dist., Kaohsiung 81157, Taiwan; kuohsun@webmail.nkmu.edu.tw; 7 Department of Animal Pharmacology, Development Centre for Biotechnology, New Taipei City 22180, Taiwan; chunming@dcb.org.tw; 8 Department of Oceanography, National Sun Yat-sen University, Kaohsiung 80424, Taiwan; lilian@mail.nsysu.edu.tw; 9 Marine Microorganism Ecological and Application Lab, Zhejiang Ocean University, Zhoushan 316004, China; xuem07@163.com; 10 Graduate Institute of Biomedical Science, China Medical University, Cancer Center Building 8F, No. 6, Hsueh-shih Road, Taichung 40477, Taiwan; 11 Center for Molecular Medicine, China Medical University Hospital, Taichung 40477, Taiwan; 12 Cancer Biology and Drug Discovery Ph.D. Program, College of Medicine, China Medical University, Taichung 40477, Taiwan

**Keywords:** benign prostate hyperplasia, *Rhodobacter sphaeroides*, probiotics, Lycogen™

## Abstract

Benign prostate hyperplasia (BPH) is one of the most common urological problems in mid-aged to elderly men. Risk factors of BPH include family history, obesity, type 2 diabetes, and high oxidative stress. The main medication classes for BPH management are alpha blockers and 5α-reductase inhibitors. However, these conventional medicines cause adverse effects. Lycogen™, extracted from *Rhodobacter sphaeroides* WL-APD911, is an anti-oxidant and anti-inflammatory compound. In this study, the effect of Lycogen™ was evaluated in rats with testosterone-induced benign prostate hyperplasia (BPH). Testosterone injections and Lycogen™ administration were carried out for 28 days, and body weights were recorded twice per week. The testosterone injection successfully induced a prostate enlargement. BPH-induced rats treated with different doses of Lycogen™ exhibited a significantly decreased prostate index (PI). Moreover, the Lycogen™ administration recovered the histological abnormalities observed in the prostate of BPH rats. In conclusion, these findings support a dose-dependent preventing effect of Lycogen™ on testosterone-induced BPH in rats and suggest that Lycogen™ may be favorable to the prevention and management of benign prostate hyperplasia.

## 1. Introduction

Many compounds extracted from microorganisms such as fungi and bacteria have been implicated in clinical treatments and healthcare. Lycogen™, derived from the extracts of the photobacterium *Rhodobacter sphaeroides* WL-APD911, is a commercial carotenoid product. The composition of Lycogen™ includes ζ-carotene, neurosporene, spheroidenone and methoxyneurosporene. ζ-carotene is the precursor of neurosporene, which is the intermediate in the biosynthesis of lycopene [1,2,3]. Carotenoids are efficient free-radical scavengers against oxidative assault; simultaneously, they are also used for medical and biotechnological purposes and as potent antimicrobial agents [4,5]. Compared to single compound lycopene, Lycogen™ is a mixture with major components that contain spheroidenone and methoxyneurosporene, which presents more potent anti-oxidant and anti-inflammatory properties, as well as less cytotoxicity [6]. Lycogen™ is the mixture and source of carotenoids, reducing the NO production and the inducible nitric oxide synthase (iNOS) expression in activated macrophages [1]. Furthermore, Lycogen™ ameliorates the inflammation of dextran sodium sulfate (DSS)-induced colitis and cisplatin-induced renal injury by reducing the expression of pro-inflammatory cytokines, TNF-α and IL-1β [2,7,8]. A more recent study showed, via a primary skin irritation test with hamsters, no irritation response from the hamster skin after 30 days of treatment with 0.2% Lycogen™ [6].

The prostate is located just below the bladder and surrounds the urethra. Benign prostate hyperplasia (BPH) is a highly prevalent urological disease in elder men and usually starts after the age of forty, further affecting about 40% of fifty-year-old men. The prevalence rate of BPH progressively increases with age and will be over 80% in octogenarians [9,10]. Pathological BPH displays an enlargement of the prostate nodules, resulting from hyperplasia of the stromal and epithelial cells, and it further results in the progressive narrowing of the urethra. Clinical symptoms comprise an increase in the frequency of urination, especially at night, a slow and weak urinary stream, and an incomplete emptying of the bladder [11,12]. BPH is also the most common cause of lower urinary tract symptoms (LUTS) and leads to a deterioration in the quality of life and sleep patterns [11].

The hyper-proliferation mechanism of the stromal and epithelial cells resulting in BPH is still unclear. Several factors such as age related hormone alteration, metabolic syndrome and growth factors, presumably contribute to the pathogenesis of BPH [13,14]. Furthermore, mounting evidence points to a connection between chronic prostatic inflammation and the cause and progression of BPH [13,15]. The treatment strategies of BPH contains 5α-reductase inhibitors, α_1_-adrenoceptor antagonists as well as anti-inflammatory agents, that are used to improve BPH and its symptoms. Unfortunately, adverse effects are known to occur from the above-mentioned pharmaceutical interventions, including dizziness, somnolence, erectile dysfunction, orthostatic hypotension and cardiovascular risks, limiting the use of these interventions in clinical practice [13,16,17,18,19].

In terms of substitutions, growing evidence and clinical trials support the implication of phytotherapy and nutraceuticals in the treatment of BPH [19,20,21]. Herbal medicines and nutraceuticals have a similar efficacy but a higher safety because of fewer side effects. In Italy, herbal medicines account for almost 50% of the medications used in the clinical treatment of BPH, whereas 5α-reductase inhibitors and α_1_-adrenoceptor antagonists account for 5% and 5%, respectively [19,21]. Plant polyphenols and its metabolites, such as isoflavonoid and equol, were showed potent anti-oxidant property to decrease prostatic neoplasia and might be beneficial effects for prostate health [22,23,24]. Lycopene is one of the nutraceuticals and can be obtained from dietary sources such as tomatoes; it is a member of the carotenoid family and has a strong antioxidant ability [19,25]. Previous case-control and cohort studies indicate that high amounts of dietary lycopene intake may lower the risk of prostate cancer and limit tumor growth and cell proliferation [26,27,28]. The meta-analysis also reports a decreased incidence of BPH diagnoses when using a lycopene supplementary; however this association is not statistically significant due to the limited follow-up periods in those studies, ranging from 3 weeks to 5 months [28].

Given the dramatic biotechnological availability of Lycogen™ and its more potent bioactivity, when compared to lycopene, we attempted to investigate the potential of the *Rhodobacter sphaeroides* extract Lycogen™ on the treatment of benign prostate hyperplasia.

## 2. Results

### 2.1. Lycogen™ Significantly Suppressed Prostate Enlargement in Testosterone Induced BPH Rats

**Figure 1:**
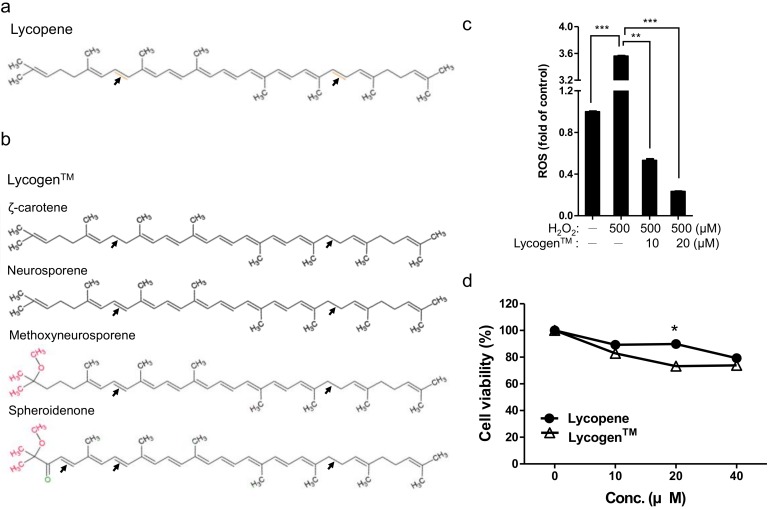
Anti-oxidative property of Lycogen™ and potency comparison of lycopene and lycogen™. The structure of lycopene (**a**) and Lycogen™ (**b**) were showed. Lycogen™ structures, including ζ-carotene, neurosporene, spheroidenone, and methoxyneurosporene were illustrated. (**c**) Detection of ROS in lycogen™-treated HK-2 cell. Histogram showing the relative fluorescence intensity of H_2_DCFDA labelling cells. Significant reduction of ROS level was observed in 10 and 20 µM lycogen™-treated HK-2 cell. (**d**) Cell viability of DU145 cell with either lycopene or lycogen™ treatment were measured by MTT assay. Histogram showing the dose-dependent decrease of lycogen™ treated DU145 cell. Significant reduction of cell viability by 20 µM lycogen™ treatment was marked. Error bars represent ± SD. * *p* <0.05, ** *p* <0.01 and *** *p* <0.001, Student’s *t*-test.

We began by testing the effect of Lycogen™, containing spheroidenone and methoxyneurosporene major components (Figure 1a,b), on anti-oxidation and cell viability. To reveal the effect of Lycogen™ on anti-oxidation, we compared the ROS level in peroxides (H_2_O_2_)-treated HK-2 cell model. Results exhibited significant higher ROS levels (3.56-fold) in the presence of 500 µM H_2_O_2_; however, the H_2_O_2_-induced ROS levels were reduced to 0.53- and 0.23-fold in 10 and 20 µM Lycogen™-treated group (Figure 1c). This dose-dependent reduction manner revealing the anti-oxidation ability of Lycogen™. To evaluate the potency of Lycogen™ and Lycopene, we compared the cell viability by using DU145 prostate cancer cell. Results of this experiment showed that Lycogen™-treated group exhibit significant lower cell viability (72.08%) compared with Lycopene-treated group (93.13%) in 20 µM (Figure 1d). Meanwhile, other treatment conditions also showed similar results (Figure 1d). Taken together, these results revealed that Lycogen™ is an anti-oxidant compound that can reduce abnormal cell proliferation.

**Figure 2:**
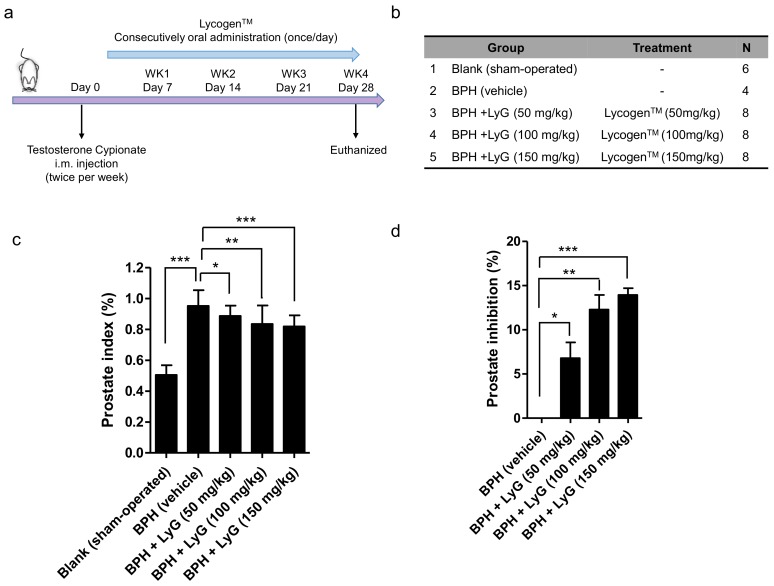
The inhibition effect of the oral administration with Lycogen™ (LyG) on BPH-induced rats. (**a**) The diagram depicted the experimental design. Benign prostate hyperplasia in rats was induced via the intramuscular injection of testosterone for 4 weeks after castration. Meanwhile, the Lycogen™ was orally supplemented for 28 continuous days in order to evaluate the treatment. (**b**) The table represents the experimental groups. Blank: the rats were sham-operated (*n* = 6); BPH: the BPH-induced rats received the solvent, pumpkin oil (*n* = 4); BPH + LyG: the BPH-induced rats received different doses of Lycogen™, including 50, 100 and 150 mg/kg (*n* = 8). (**c**) The effect of the Lycogen™ administration on the prostate weight in BPH-induced rats. The prostate index (%) represented the prostate wet weight to body weight ratio. (**d**) The prostate inhibition percentage, when compared to BPH-induced rats, was shown. The data were presented as the mean ± SD. Blank: sham-operated; BPH: BPH-induced rats; BPH + LyG: BPH-induced rats combined with a Lycogen treatment (50, 100 and 150 mg/kg). Error bars represent ± SD. * *p* <0.05, ** *p* <0.01 and *** *p* <0.001, Student’s *t*-test.

We then test the effect of Lycogen™ on testosterone-induced BPH rats. In our pilot studies, the safety dose of Lycogen™ was determined according to the standard toxicological screening protocol [29]. Results showed that the safety dosage of Lycogen™ is 150 mg/kg/day for biological and behavioral endpoints, which also showed without any genetic toxicity in their pups. The Sprague-Dawley male rats (200–250 g) were castrated or received a sham-operation procedure followed by an injection with testosterone to induce BPH; meanwhile, they also received an oral administration of pumpkin oil (vehicle group) or an indicated dose of Lycogen™ (50, 100 and 150 mg/kg) for 28 days (Figure 2a,b). To determine the effect of Lycogen™ on prostate enlargement, we analyzed the prostate index (prostate weight/bodyweight) after treatments with different Lycogen™ doses. BPH-induced groups, including the vehicle and Lycogen treatments, displayed a significant increase in the prostate size, prostate weight, and prostate index compared with the blank (sham-operated rats) treatments (Table 1). The mean prostate weight of BPH-induced rats was 3.89 g, which was significantly higher than the mean prostate weight of sham-operated rats (2.294 g). This significant increase of prostate weight also reflected to the increase of prostate index (~1.89-fold) in the BPH-induced rats (Figure 2c). These data demonstrating that our BPH model was successfully established.

**Figure 3:**
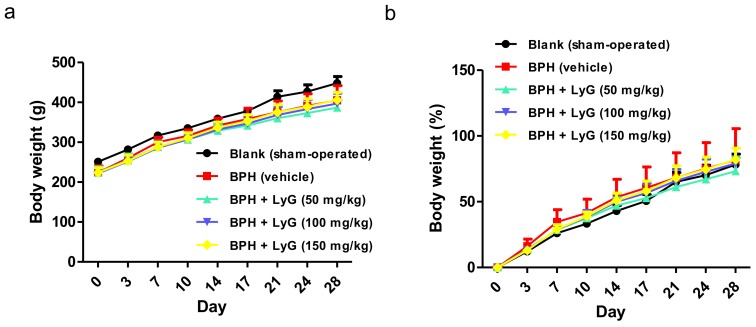
Body weight of BPH-induced rats who had an oral administration of Lycogen™ (LyG). (**a**) The mean body weight of each group and (**b**) the mean body weight normalized to the control of each group that were exhibited for BPH-induced rats during the period of Lycogen™ administration. The data were presented as mean ± SD. Blank: sham-operated; BPH: BPH-induced rats; BPH + LyG: BPH-induced rats combined with a Lycogen™ (LyG) treatment (50, 100 and 150 mg/kg).

The BPH-induced rats treated with Lycogen™ displayed a dose-dependent inhibition manner in the prostate index, compared with the vehicle group (Figure 2c). The prostate weight ratio of the administration group treated with a higher amount (150 mg/kg) of Lycogen™ was significantly lower than that of the BPH rats without treatment (Figure 2c). The percentage of prostate inhibition was found to be 6.8%, 12.3% and 13.9% for the administration of 50, 100 and 150 mg/kg of Lycogen™, respectively (Figure 2d and Table 1). A dose-response reduction in prostate enlargement was displayed in the BPH-induced rats who received an increasing dose of Lycogen™. Additionally, the testosterone injection and further Lycogen™ administration did not profoundly influence the body weight during the experimental period (Figure 3a,b). The body weights were comparable in all groups at the end of the experiment. This suggested that Lycogen™ may potentially attenuate BPH at high dose levels such as 150 mg/kg.

### 2.2. Lycogen™ Dramatically Reduced Prostate Lesions in Testosterone Induced BPH Rats

**Figure 4:**
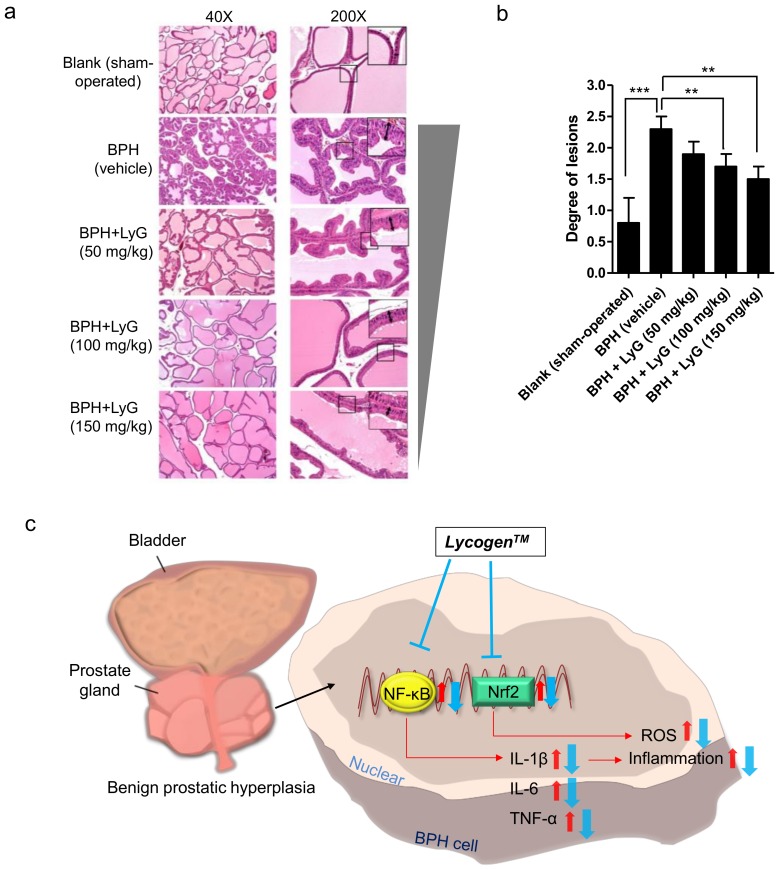
The histopathology of the prostate in the BPH-induced rats after being treated with Lycogen™. (**a**) A comparison of different doses of Lycogen on prostate hyperplasia according to H&E staining of the prostate sections. The H&E staining sections were photographed by microscope at 40- and 200-fold magnifications. The thickness of the epithelial was marked with double-headed arrows. The enlarged images represented the area indicated using a black square. The grey triangle represented a dose-dependent manner of the inhibition effect of Lycogen™ in BPH tissues. (**b**) The quantification of H&E staining sections was done according to a five grade score, included score 1: reflects minimal hyperplasia (<1%); score 2: reflects slight hyperplasia (1–25%); score 3: reflects moderate hyperplasia (26–50%); score 4: reflects moderately severe/high hyperplasia (51–75%); score 5: reflects severe/high hyperplasia (76–100%). The data were presented as a mean ± SEM. Blank: sham-operated; BPH: BPH-induced rats; BPH + LyG: BPH-induced rats combined with a Lycogen treatment (50, 100 and 150 mg/kg). Error bars represent ± SEM. ** *p* <0.01 and *** *p* <0.001, Student’s *t*-test. (**c**) Postulated mechanisms of how Lycogen may reduce BPH. References utilized in this composite figure include [1,2,6,7,30,31,32]. Red arrow represented up-regulation of gene/protein level; blue arrow represented down-regulation of gene/protein level. Blue T bar represented the inhibition effect of Lycogen™.

Next, we examined whether Lycogen™ affects BPH progress by using a histopathological analysis. The histological morphology of the prostate tissue was examined via H&E staining. The prostatic epithelial cells in the blank group were flattened, cuboidal and regular in size (Figure 4a). Compared with the blank group (sham-operated rats), the epithelial cell layer in the BPH-induced rats were larger, and the epithelium became thicker. Numerous papillae projecting into the lumen formed and further decreased the glandular luminal area (Figure 4a). The BPH-induced rats treated with Lycogen™ displayed some features of prostate hyperplasia but were apparently ameliorated in a Lycogen™ dose-dependent manner (Figure 4a). The thinner epithelium height and reduction in epithelial cell proliferation were visibly observed in the Lycogen™ (100 mg/kg) and Lycogen™ (150 mg/kg) groups (Figure 4a). The histopathological change of each group was also scored and semi-quantitative (Figure 4b). The prostate hyperplasia in the BPH-induced rats was significantly improved after an administration with an increased dosage of Lycogen™.

## 3. Discussion

In the present study, we investigate the effects of Lycogen™, a novel compound extracted from *Rhodobacter sphaeroides*, on benign prostatic hyperplasia (BPH) in rats induced by injection with testosterone after castration. From these results, we suppose that the prostate enlargement was attenuated in BPH-induced rats simultaneously supplied with Lycogen™, achieving the prevention when treated at a high dose.

BPH, the fourth most frequently diagnosed disease in elderly men, occurs with aging, and the degree and progress of BPH is different from individual to individual. The cost of intervention and treatment of BPH is estimated at approximately four billion dollars annually in the United States [33]. Although the pathogenesis of BPH is not fully understood, several factors, including hormone alteration, growth factors, metabolism syndromes and prostatic inflammation, have been demonstrated to contribute to the development of BPH. Moreover, prostatitis and oxidative stress also induce the inflammatory cascade related to the nuclear factor κ-B (NF-κB) and to iNOS overexpression and cytokines production [12]. The pro-inflammatory cytokines, TNF-α and IL-1β, are well known and play a critical role in inflammatory processes. Both of them have been used as markers of prostatic inflammation in cases of chronic prostatitis [34]. The expression of IL-1β is up-regulated in the inflammatory prostatic cell line [30]. Hence, one strategy for medical therapy against BPH is the use of anti-inflammatory drugs including nonsteroidal anti-inflammatory drugs (NSAIDs), phytotherapeutic agents and vitamin D receptor agonists [13]. However, along with the 5α-reductase inhibitors and α_1_-adrenoceptor antagonists, NSAIDs targets on the inhibition of COX, COX-1 and COX-2 also cause adverse effects that limit their use in clinical treatments [16]. Phytotherapy, more safe and with similar effectiveness, is an alternative choice for treating prostate inflammation [13]. Numerous extracts or seeds of different plants including *Glycine max* (Soybean), *Secale cereale* (rye), *Serenoa repens* (saw palmetto), *Prunus africanum* (pygeum), lycopene and *Serenoa repens* (HESr) have been investigated for their anti-inflammatory property and recommended for use in the treatment of BPH [13,19,24]. 

Carotenoids are secondary metabolites synthesized by plants, fungi, algae, and bacteria [35]. Because of antioxidant and Vitamin A activity, carotenoids are widely applied in nutraceuticals, pharmaceuticals, poultry, food and cosmetics. The current commercial market value of these carotenoids is growing to $1.8 billion in 2019 with an annual growth rate of 2.3% [36]. Although a wide variety of carotenoids is found, only a few have been commercially available. The plant-based sources of carotenoid production are limited which resulted in low yields and high product costs [35]. *Rhodobacter sphaeroides* belongs to photobacteria, which usually serve as probiotics in aquiculture industry. *R. sphaeroides*, WL-APD911, whose carotenoid 1,2-hydratase (crtC) gene was mutated and leading to produce a new carotenoid with higher antioxidant ability [1]. Lycogen™ is a commercial carotenoid product extracted from WL-APD911, which have many advantages over plant carotenoids [36,37,38,39]. One such advantage is fermentation; the growth rate of microbe is faster than plant. The other enduring strength of microbes is that they are more easy to manipulate and culture than plants [4,40]. These advantages of microbes provides a more efficient process for large-scale production and low production cost.

The composition of Lycogen™ includes ζ-carotene, neurosporene, spheroidenone and methoxyneurosporene. ζ-carotene is the precursor of neurosporene, which is the intermediate in the biosynthesis of lycopene [41]. Several in vitro and in vivo studies have documented that lycopene is probably helpful in preventing BPH [42,43]. Lycopene, a member of the carotenoids family, displays singlet oxygen quenching activity and exhibits a strong antioxidant capacity [43]. Carotenoids, like ζ-carotene and lycopene, are known to activate these reaction by directly quenching singlet oxygen and free radicals [44], or indirectly by up-regulating the phase II detoxifying enzymes through electrophile response element/antioxidant response element (EpRE/ARE) transcription system [45]. Nuclear-factor-erythroid 2-related factor 2 (Nrf2) and nuclear factor kappa-light-chain-enhancer of activated B cells (NF-κB) play vital roles in defending cellular oxidative stress and anti-inflammation responses [23,46,47]. Lycopene were showed to transcriptionally activate antioxidant enzymes, such as the phase II enzymes NAD(P)H: quinone oxidoreductase (NQO1), glutamate cysteine ligase (GCL) and superoxide dismutase (SOD), through Nrf2 and NF-κB- mediated pathways [48,49]. Transcription factors like NF-κB and Sp1 are consequently suppressed, thus, modulating target gene expression and then blocking cell cycle progression. Furthermore, lycopene treatment also strongly interfere IGF-1 receptor signaling, Ras signaling and MAPK (ERK, JNK, p38) and PI3K/Akt [50]. Taken together, the antioxidant enzyme system helps cells against oxidant stress, lipid oxidation, DNA damage and apoptosis [51]. Thus, avoiding antigen releasing and preventing the production of inflammatory cytokines [52]. Accumulating evidence also showed that anti-oxidant and anti-inflammation compounds, such as isoflavone and equol, mediated cellular responses through the same mechanism [46].

The anti-oxidative and anti-inflammatory properties of Lycogen™ have been demonstrated when mounting our prior investigations [1,2,7,31,32,49,53]. Lycogen™ treatment was demonstrated to inhibit NO production and inducible nitric-oxide synthase (iNOS) expression in activated macrophages [1]. Lycopene also inhibited cell proliferation in normal prostate epithelial cells and the promotion of apoptosis in hyperplasia prostate tissue [19,54,55]. Additionally, the production of TNF-α and IL-1β is significantly inhibited after oral administration of Lycogen™ in mice with DSS-induced colitis [2] or renal injury [7]. Our pervious study also demonstrated that Lycogen™ treatment can reduce the UVA-induced NF-κB level [49]. Since Lycogen™ and lycopene share the same biosynthesis pathway, we believed that Lycogen™ shared similar cellular mechanisms with lycopene [53,56]. The anti-inflammatory and anti-oxidative abilities that have been attributed to Lycogen™ may partially result from the effects of lycopene; however, we have shown that Lycogen™ significantly inhibit the cell viability of prostate cancer cell compared with Lycopene (Figure 1d). Our pervious study also demonstrated that spheroidenone [57] and methoxyneurosporene [58] are major components of Lycogen™ which presented with more potent antioxidative activity and less cytotoxicity than the single compound, lycopene [6,49]. Taken together, these data suggest that Lycogen™ has more potency than lycopene for prostate health.

Based on our studies and reports from other research groups, the possible mechanisms of how Lycogen™ improves BPH have been reviewed and summarized (Figure 4c). Lycogen™ may inhibit the development of BPH through Nrf2- and NF-κB-mediated signaling pathways [32,53]. Further works are required to elucidate how Lycogen™ contributes to the reduction of BPH and the clarification of a therapeutic potential, as well as the prevention of BPH in humans.

## 4. Materials and Methods

### 4.1. Lycogen™ and Animals

The patent strain (*R. sphaeroides* WL-APD911) was created by Asia-Pacific Biotech Developing InC. (Kaohsiung, Taiwan). The strain was cultured in broth. After harvesting, the bacteria were concentrated by centrifugation followed by washing with ethanol. After extraction with acetone, the bacteria residue was centrifuged at 7500 rpm for 5 min. The supernatant was collected and filtered with filter paper and a 0.2 µm filter into a flask. The appearance of the supernatant was dark red. The supernatant was incubated in a 55 °C oven to remove the acetone completely. This extract from *R. sphaeroides* WL-APD911 was named Lycogen™, which is an available and commercial product. Lycogen™ was dissolved in pumpkin oil (Sigma-Aldrich, St. Louis, MO, USA). All the male *Sprague-Dawley rats* were purchased from BioLASCO in Taiwan. All rats were housed under regular and stable conditions of temperature and a 12 h light-dark cycle. The animal use protocol was approved by the Institutional Animal Care and Use Committee (IACUC) of the Developmental Center for Biotechnology. All the experiments were performed according to the international guidelines and regulations.

### 4.2. Measurement of Cell Viability

The effect of Lycogen™ on cell viability was tested by 3-(4,5-dimethylthiazol-2-yl)-2,5-diphenol tetrazloium bromide (MTT) assay [49]. DU145 cells (1 × 10^4^ cells/well) were seeded in 24-well plates with Eagle’s Minimum Essential Medium (EMEM) at 37 °C for 24 h, and the medium was replaced by Lycogen™ containing EMEM for another 48 h. Then, 20 µL MTT (5 mg/mL) was added and incubated for 1 h at 37 °C. The reaction was stopped by replacing the medium with 200 µL DMSO for 15 min. The absorbance at 570 nm was then recorded using a microplate reader (FLUOstar OPTIMA, BMG Labtech GmbH, Offenburg, Germany).

### 4.3. Measurement of Intracellular ROS

The intracellular accumulation of ROS was determined using the fluorescent probe 2′,7′-difluorodihydrofluorescein diacetate (H_2_DCFDA, Sigma-Aldrich), which is commonly used for detecting cellular H_2_O_2_. HK-2 cells (8 × 10^4^) were treated with 0.5 µM ABT-751 (Sigma-Aldrich) and then incubated with the probe (10 µM) in dark at 37 °C for 30 min. Cells were washed with PBS and then analyzed with flow cytometry (BD Biosciences, San Jose, CA, USA). Fluorescence signals at the excitation wavelength of 488 nm and emission wavelength of 530 nm were recorded.

### 4.4. Establishment of Experimental BPH Model

The animal model, prostate hyperplasia rats, was established according to the methods previously described and slightly modified [59,60]. Young adult male Sprague-Dawley rats at 6–7 weeks of age underwent castration or a sham-operation. Briefly, the sham-operation means that the scrota of 6 rats were cut open and sewed up without slicing off the testis. After castration, the rats were induced by intra-muscular injection of Testosterone Cypionate (10 mg/kg; Tai Yu Chemical & Pharmaceutical Co., Ltd., Hsinchu, Taiwan) twice per week [3,61]. 

Lycogen™ was dissolved in pumpkin seed oil that administered orally to rats via metal tube feeding (gavage procedure). In brief, the distance from the oral cavity to the end of the xyphoid was measured, which is the distance when the tube be inserted into the esophagus. Lycogen™ containing oil was loaded in 1 mL feeding syringe which connected with a metal feeding tube. Then, the oil was smoothly injected into the esophagus. To investigate the optimal dosage and therapeutic effect of Lycogen™, when beginning to induce BPH (Day 0), rats were also supplemented orally for 28 consecutive days by gavage at the same time (Day 0–28). The control group was treated with the solvent, pumpkin seed oil. According to the treatment, they were assigned to the following groups: (1) Blank group (sham operation); (2) BPH + vehicle group: the BPH-induced rats who received the solvent, pumpkin oil; (3) BPH + LyG (50 mg/kg): the BPH-induced rats who received Lycogen™ (50 mg/kg); (4) BPH + LyG-L (50 mg/kg); (5) BPH + LyG-M (100 mg/kg); (6) BPH + LyG (150 mg/kg). The body weight (BW) of each rat was monitored and recorded twice per week during the treatment period.

### 4.5. Assessment of Prostate Index (PI) Percentage and Prostate Inhibition Percentage

After 28 days of treatment, the rats were anesthetized by intraperitoneal injection with pentobarbital (60 mg/kg) and the prostate was immediately removed and weighed. The mean BWs and prostate index (PI) were calculated for each group. The equation of the PI index and prostate inhibition percentage were listed:

Prostate index percentage = (prostate wet weight/body weight) × 100%

Prostate inhibition percentage = 100 − [(prostate wet weight of BPH rats treated with Lycogen™/prostate wet weight of BPH rats) × 100]

### 4.6. Histological Examination

The weighted prostate tissues were fixed in PBS containing 10% paraformaldehyde followed by processing for their embedding in paraffin wax. The embedded tissues were sectioned into a thickness of 3–5 µm and stained with hematoxylin and eosin (H&E) for histological analysis. The histopathological scoring and assessment is performed via the qualitative and quantitative analysis described in [62]. The evaluation is divided into 5 grades: (1) a score of 1: reflects minimal hyperplasia (<1%); (2) a score of 2: reflects slight hyperplasia (1–25%); (3) a score of 3: reflects moderate hyperplasia (26–50%); (4) a score of 4: reflects moderately severe/high hyperplasia (51–75%); (5) a score of 5: reflects severe/high hyperplasia (76–100%). The histopathological changes of each group were scored and semi-quantitative.

### 4.7. Statistical Analysis

All data were expressed as the mean ± standard deviation (SD) or mean ± standard error of the mean (SEM). The Student *t*-test was used to determine the differences between each group for making comparisons. Differences were considered to be statistically significant at *p* <0.05. 

## 5. Conclusions

Lycogen™ shows a dose-dependent preventing effect of on testosterone-induced BPH in rats, which suggests that Lycogen™ may be advantageous to the prevention and management of benign prostate hyperplasia.

## Figures and Tables

**Table 1:** Effect of Lycogen^TM^ (LyG) products on prostate enlargements in testosterone induced rats.

Group	Dose (mg/kg)	PW (g)	Inhibition (%)	Prostate Index	Inhibition (%)
Blank (sham-operated)	0	2.294 ± 0.088		0.504 ± 0.03	
BPH (vehicle)	0	3.892 ± 0.239		0.952 ± 0.05	
LyG-50 mg/kg	50	3.482 ± 0.095	10.5% ***	0.887 ± 0.02	6.8% ***
LyG-100 mg/kg	100	3.338 ± 0.109	14.2% ***	0.835 ± 0.04	12.3% ***
LyG-150 mg/kg	150	3.375 ± 0.117	13.3% ***	0.819 ± 0.03	13.9% ***

Values are expressed as mean ± SEM, *n* = 8; PW: Prostate weight; *** Significant difference in compared to BPH (*p* < 0.001).
